# Depressive and Anxiety Symptoms in Community-Dwelling Women in Rural Areas of Greece in the Post-COVID-19 Pandemic Era

**DOI:** 10.3390/jcm13195985

**Published:** 2024-10-08

**Authors:** Vaios Peritogiannis, Alexandra Mantziou, Nikolaos Vaitsis, Stamatina Aggelakou-Vaitsi, Maria Bakola, Eleni Jelastopulu

**Affiliations:** 1 Mobile Mental Health Unit of the Prefectures of Ioannina and Thesprotia, Society for the Promotion of Mental Health in Epirus, 44445 Ioannina, Greece; 2 Primary Healthcare, 40300 Farsala, Greece; 3 Department of Public Health, School of Medicine, University of Patras, 26500 Patras, Greece

**Keywords:** anxiety, depression, depression, anxiety, stress Scale-21, elderly, rural areas, women

## Abstract

**Background/Objectives**: Depressive and anxiety syndromes are associated with elevated disability and are more prevalent in women. Data on the prevalence of depressive and anxiety disorders in the rural context are limited and contradictory. It is relevant to study common mental disorders in rural areas in the most vulnerable population of women, particularly in the post-COVID-19 pandemic era. **Methods**: This is a cross-sectional study that was conducted in two primary healthcare sites in the rural region of Farsala, Central Greece after the obviation of all restrictive measures that had been posed due to the COVID-19 pandemic. All consecutive female patients that attended the study sites for any non-emergent reason were asked to participate in the study. For the recording of symptoms of depression and anxiety, the self-reported Depression, Anxiety, Stress Scale-21 (DASS-21) was used. **Results**: The study sample consisted of 129 women. The majority of participants were >50 years, with 27.9% being older adults. A small percentage (13.2%) suffered a chronic physical disease. A large proportion of the sample, slightly exceeding 40%, reported clinically relevant symptoms of anxiety, whereas a lower percentage of women with depressive symptoms was detected (17.1%). Symptoms of anxiety and depression were found to be interrelated, while a number of sociodemographic variables were associated with both, such as older age, education (primary), living status (alone, OR 123.5; 95% CI: 7.3–2098.8 for anxiety; OR 3.5; 95% CI: 1.3–9.8 for depression), employment (not working, (OR 0.157; 95% CI: 0.06–0.41 for anxiety; OR 0.08; 95% CI: 0.01–0.62 for depression) and the history of a chronic disease (OR 33.8; 95% CI: 4.3–264.7 for anxiety; OR 37.2; 95% CI: 10–138.1 for depression). Self-rated financial status was not related to symptoms of anxiety or depression. **Conclusions**: The study highlights the importance of inquiring for symptoms of depression and anxiety in women attending the rural primary care setting. The use of valid and reliable self-reported instruments that are easy to administrate may be helpful in this regard.

## 1. Introduction

The Global Burden of Disease 2019 report confirmed that mental disorders remained among the leading causes of burden worldwide. Among mental disorders, the so-called common mental disorders, such as depressive and anxiety syndromes, have been associated with early and elevated disability in patients and contribute significantly to the global burden of disease [1,2]. Common mental disorders may be more prevalent in women; for instance, the point prevalence of depressive symptoms in women has been found to be twice as high as in men [3]. Accordingly, the burden of depressive and anxiety disorders was greater in females than males [1].

With regard to the correlation of place of residence with common mental disorders, research findings are contradictory. A previous study in the USA found that the prevalence of depression was significantly higher in residents of rural areas compared to urban areas [4]. Similarly, a systematic review on the epidemiology of depression in China found that rural residents had a greater risk of depression than urban residents [5]. On the contrary, according to a recent meta-analysis, urban residency was significantly associated with a higher prevalence of depression in developed but not in developing countries [6]. A recent study in the U.S.A. revealed no differences between patients living in rural and urban communities in terms of the rates of psychiatric disorders, severity of psychiatric symptoms and functional impairment. The authors suggested that the needs for mental health treatment are similar in urban and rural areas and a possible explanation for the occasionally reported rural–urban differences in the rates of psychiatric disorders could be the access to mental healthcare, which is easier in urban locations [7]. Furthermore, a recent meta-analysis on the prevalence of depression and anxiety among young persons in Australia revealed no differences between rural and urban residents [8]. Yet, in India, a higher rate of depression among rural elderly than in urban elderly has been consistently reported [9], including older adults with multimorbidity [10].

The COVID-19 pandemic in early 2020 resulted in radical changes in everyday living globally, including social distancing, isolation and limited socioeconomic activity. The acute effects of the pandemic and the subsequent restrictive measures on the mental health of the population have been extensively studied in Western countries. Several psychological reactions to the pandemic have been recorded, such as distress, anxiety, fear, frustration, anger and depression [11,12,13]. The psychological impact of the COVID-19 pandemic on the Greek population has been highlighted by several studies that reported rates of depressive and anxiety symptoms ranging from 12.9 to 22.8% and 20 to 77.4%, respectively [14,15,16]. A previous history of a common mental disorder and current treatment with psychiatric medication has been associated with the development of anxiety and depressive symptoms.

Regardless of the extent that rural dwellers are affected by common mental disorders, they are less likely than their urban counterparts to seek or receive mental health services [17] due to the well-documented mental health disparities between rural and urban areas [18]. It is thus relevant to explore symptoms of depression and anxiety in the rural context, and this is particularly the case of the most vulnerable part of the population in this regard, which are women. The study of such symptoms in rural populations may be even more relevant after the collective stressful event of the COVID-19 pandemic. Accordingly, the present study aimed to explore symptoms of depression and anxiety in rural women attending the primary healthcare setting in the post-pandemic era; that is, after the quarantine intervals that were posed as restrictive measures due to the pandemic. Another study objective was to explore the co-occurrence of anxiety and depressive symptoms; finally, the study also aimed to address the correlations of anxiety and depressive symptoms with sociodemographic and clinical variables.

## 2. Materials and Methods

### 2.1. Study Setting and Design

This is a cross-sectional study that was conducted in two primary healthcare sites in the rural region of Farsala, Central Greece over a 2-month period (January–February 2023) after the lifting of all the restrictive measures that had been imposed due to the COVID-19 pandemic. All of the female patients that attended the study sites for any non-emergent reason (that is, a sub-acute clinical problem, prescription refill, scheduled examination, etc.) were asked to participate in the study and were informed about the study procedures. To be included in the study, the women had to be older than 18 years and have the ability to read and understand the Greek language. The exclusion criteria were a diagnosis of cancer, rheumatoid disease, or a receipt of medication for a diagnosed mental disorder. A total of 210 women that visited the primary care study sites during the study period were approached for participation. Of those, 26 refused to participate, whereas 55 met the exclusion criteria. The final sample of the study consisted of 129 women. All the procedures of the study had been approved by the Ethics Committee of the Hellenic Open University (75/13-12-2022). Written informed consent was obtained by all participants.

### 2.2. Data Gathering

All participants had to fill a form with demographic information regarding age; education (primary, which is up to 9 years; secondary, which is 10–12 years; and tertiary, which is >12 years); accommodation status (living with someone or alone); employment (working vs. not working, including pensioners); financial status (poor/moderate/good, according to self-ratings); and a history of a chronic disease. 

For the recording of symptoms of depression and anxiety, the Depression, Anxiety, Stress Scale-21 (DASS-21) was used. The DASS-21 is a self-reported instrument measuring anxiety, depression and stress in the reference period of the past week. The Depression scale assesses symptoms such as dysphoria, hopelessness, self-worthlessness and lack of interest; the Anxiety scale comprises items evaluating somatic symptoms, situational anxiety and anxious affects; the Stress scale measures symptoms such as difficulty relaxing, agitation, irritability and impatience [19]. 

The Greek version of the DASS-21 consists of 21 items, which measure three emotional states that are depression, anxiety and stress. Each item is rated according to the Likert scale from 0 (Did not apply to me at all) to 3 (Applied to me very much or most of the time). Each subscale consists of 7 items (questions) and the total score for each subscale is calculated by the sum of scores, multiplied by 2 (from 0 to 42). In the present study, the subscales of depression and anxiety were employed. Scores <10 and <8 were rated as “no depression” and “no anxiety” in the respective subscales. 

The original 42-item version of the DASS has been translated in Greek and validated in the general population and in psychiatric patients [20]. The results have shown that the scale has excellent internal validity with Cronbach’s alpha coefficients ranging between 0.90 and 0.97 for the three scales and the total scale. Also, the scale showed good convergent and discriminant validity. More recently, the psychometric properties of the Greek DASS-21 version have been validated in the Greek population and it was shown that the Greek DASS-21 can be used as a reliable and valid instrument for the measurement of depression, anxiety and stress in the Greek population [21]. The study of the dimension of stress was omitted in the present study which focused on clinically relevant symptoms of depression and anxiety. 

### 2.3. Statistical Analysis

The analysis was carried out with descriptive statistics. Categorical variables are presented as absolute and relative frequencies (n, n%). To investigate the relationship between two categorical variables, the chi-square test was used. Cramer’s V was used to measure the effect size for the chi-square test of independence. In the case of a 2 × 2 contingency table, Cramer’s V is equal to the absolute value of Phi coefficient. Additionally, in the cases of 2 × 2 tables, we used an odds ratio (OR) with a 95% confidence interval (CI) to measure the effect size. The level of statistical significance, the *p*-value, was set equal to 0.05. Data analysis was performed with IBM SPSS 25 (Statistical Package for Social Sciences, vs 25).

## 3. Results

The study sample consisted of 129 women. The clinical and demographic characteristics of the sample are presented in Table 1. The majority of participants were >50 years, whereas 27.9% (n = 36) were older adults. Most had up to secondary education and one in four (n = 32) had tertiary education. Most participants were living with someone and only 17.8% were living alone. Forty-one (31.8%) were employed and equal number of participants (n = 46, 35.7%) rated their financial status as modest or poor. Only 17 participants (13.2%) suffered a chronic physical disease. 

According to the ratings in the DASS-21, 52 of the participants (40.3%) displayed clinically relevant anxiety symptoms, whereas 22 participants (17.1%) were found to have clinically relevant depressive symptoms. The chi-square test revealed a statistically significant relationship between anxiety and depression [χ^2^(1) = 39.275, *p* < 0.001]. The correlation was large; Cramer’s V = 0.552. Women with anxiety reported a higher rate of depression than women without anxiety; 42.3% versus 0.0%. Similarly, women with depression reported a higher rate of anxiety than women without depression; 100% versus 28.0%.

The chi-square test revealed a statistically significant correlation between anxiety and age [χ^2^(3) = 16.901, *p* = 0.001]. The correlation was large; Cramer’s V = 0.362. Specifically, the rate of anxiety in the 18–35 age range was calculated at 18.2%, in the 36–50 age range 28.1%, in the 51–65 age range 38.5% and in participants >65 years 66.7%. Of all the combinations of age categories, only women aged >65 recorded a statistically significant higher rate of anxiety than women in the age range 18–35 and 36–50. The chi-square test also revealed a statistically significant relationship between anxiety and educational level [χ^2^(2) = 9.455, *p* = 0.009]. The correlation was moderate; Cramer’s V = 0.271. A decreasing trend in the anxiety rate as the educational level increases was observed. More specifically, the anxiety rate in the educational category “Primary Education” was calculated at 75.0%, in the “Secondary Education” category 37.0% and in the “Tertiary Education” category 31.3%. Of all the combinations of educational levels, only women who received education up to the primary level registered a statistically significant higher rate of anxiety than women belonging to the other educational levels. With the use of chi-square test a statistically significant relationship between anxiety and living status [χ^2^(1) = 41.448, *p* < 0.001] was revealed. The correlation was large, with Cramer’s V = 0.567 and (OR (Alone/Cohabitation) 123.5; 95% CI: 7.3–2098.8). In particular, the percentage of anxiety in women who live alone is statistically higher (100.0%) than that of women who live with at least one other person (27.4%). The chi-square test revealed a statistically significant correlation between anxiety and employment [χ^2^(1) = 16.468, *p* < 0.001]. The correlation was moderate, with Cramer’s V = 0.357 and (OR (Working/Not working) 0.157; 95% CI: 0.06–0.41). In particular, the percentage of anxiety among women who are not employed including those receiving a pension is statistically higher (52.3%) than those who work (14.6%). The chi-square test did not reveal a statistically significant correlation between clinically relevant anxiety and financial status [χ^2^(2) = 0.855, *p* = 0.652]. The correlation was small; Cramer’s V = 0.081. The percentage of anxiety in women who rated their financial status as poor, moderate, or good was found to be 45.7%, 37.0% and 37.8%, respectively. Finally, the chi-square test revealed a statistically significant relationship between anxiety and chronic diseases [χ^2^(1) = 23.561, *p* < 0.001]. The correlation was moderate; Cramer’s V = 0.427 and (OR (chronic diseases/no chronic diseases) 33.8; 95% CI: 4.3–264.7). Anxiety in women who suffer at least one chronic disease was statistically higher (94.1%) than in women who had no history of a chronic disease (32.1%). The results are summarized in Table 2.

Correlations of clinically relevant depressive symptoms with the examined clinical and demographic variables were almost identical with the aforementioned findings (Table 2). The chi-square test revealed a statistically significant correlation between depression and age [χ^2^(3) = 21.460, *p* < 0.001]. The correlation was large; Cramer’s V = 0.408. More specifically, the rate of depression in the 18–35 age category was calculated at 9.1%, in the 36–50 category 6.3%, in the 51–65 category 7.7% and in the >65 category 41.7%. Of all combinations of age categories, only women aged >65 years reported a statistically significant higher rate of depression compared to women in each of the other age categories. Also, the chi-square test revealed a statistically significant relationship between depression and educational level [χ^2^(2) = 9.682, *p* = 0.008]. The correlation was moderate; Cramer’s V = 0.274. The percentage of depression in the educational level category “Primary Education” was calculated at 43.8%, in the “Secondary Education” category 14.8% and in the “Tertiary Education” category 9.4%. Of all the combinations of educational levels, only women who received education up to primary level reported a statistically significant higher rate of depression than women belonging to the other educational levels. With regard to the association of self-rated depressive symptoms with living status, the chi-square test revealed a statistically significant correlation [χ^2^(1) = 6.219, *p* = 0.013]. The correlation was small; Cramer’s V = 0.220 and (OR (Alone/Cohabitation) 3.5; 95% CI: 1.3–9.8). In particular, the rate of depression among women who live alone was statistically higher (34.8%) than the corresponding rate among women who live with at least one person (13.2%). With the use of the chi-square test, a statistically significant relationship between depression and employment [χ^2^(1) = 9.076, *p* = 0.003] was revealed. The correlation was small; Cramer’s V = 0.265 and (OR (Working/Not working) 0.08; 95% CI: 0.01–0.62). More specifically, the rate of self-rated depression among women who do not work was statistically higher (23.9%) than the corresponding rate among women who work (2.4%). No statistically significant correlation between self-rated depression and financial status [χ^2^(2) = 0.333, *p* = 0.847] was found with the use of the chi-square test. The correlation was small; Cramer’s V = 0.051. The percentage of depression among women who rated their financial status as poor, moderate or good was found to be 19.6%, 15.2% and 16.2%, respectively. Finally, the chi-square test revealed a statistically significant relationship between depressive symptoms and chronic diseases [χ^2^(1) = 48.866, *p* < 0.001]. The correlation was large; Cramer’s V = 0.615 and OR (chronic diseases/no chronic diseases) 37.2; 95% CI: 10–138.1). In particular, the rate of depression in women who had at least one chronic disease was statistically higher (76.5%) than the corresponding rate among women who had no history of a chronic disease (8.0%).

## 4. Discussion

The present study adds to a limited literature as one of the few studies that addresses symptoms of anxiety and depression in the Greek rural context, and the first with a specific focus on women. It appeared that a large proportion of primary care attending women, slightly exceeding 40%, reported clinically relevant symptoms of anxiety, whereas a lower but notable percentage of women with depressive symptoms was detected (17.1%). Symptoms of anxiety and depression were found to be interrelated, while a number of sociodemographic variables were associated with both, such as older age, education (primary), living status (alone), employment (not working) and the history of a chronic disease. Self-rated financial status was not related to symptoms of anxiety or depression. 

In a previous Greek study that assessed depressive symptomatology in persons attending primary healthcare (56% women, mean age >65 years), a total rate of 32.6% was found. However, this study did not exclude patients with chronic rheumatic diseases or those receiving psychotropic medication [22]. In a study in rural India [23], a similar rate of depression (15%) was recorded in a female community-based sample aged 15 to 59 years with the use of the DASS-21. The reported anxiety rates were almost four times lower than the present study (10.6% vs. 40.3%). Another population-based study in rural South–Central Texas, U.S.A., reported a rate of probable depression 18.1% or 10% depending on the instrument used for symptom assessment, with women displaying higher rates than men [24]. Differences in sampling and used instruments along with cultural diversity could probably account for the observed differences in the prevalence of depressive and anxiety symptoms in rural populations across countries. Furthermore, the Indian study used a community-based sample of a wider age range compared to a clinical sample of mostly older adults in the present study. This likely also contributes to the difference in prevalence rates.

The present study recorded a significant correlation between depression and anxiety, meaning that participants with depressive symptoms were more likely to display anxiety symptoms as well, and vice versa. Previous research has documented that anxiety and depressive disorders commonly coexist [25,26] and the presence of such comorbidity increases medical utilization and may be associated with chronicity, slower recovery, increased recurrence rates and psychosocial disability [27].

Elderly women displayed more depressive and anxiety symptoms, compared to younger ones in the present study. The statistical analysis revealed an association of anxiety with age, ranging from 18.2% in ages 18–35 up to 66.7% in elderly participants, indicating that the older the person, the more anxiety they have. The pattern of depressive symptoms was different across the age-range of participants, as the prevalence of clinically relevant symptoms was somewhat similar in ages 18–35, 36–50 and 51–65 (9.1%, 6.3% and 7.7%, respectively), to increase significantly (41.7%) in older adults. According to a previous study involving older adults attending day care centers for older people in Southwest Greece, the rates of depression were high (48.1%) [28]. Although the results of that study are somewhat similar to the results of the present study (41.7% depressive symptoms in older participants), several differences should be considered. First, the study by Argyropoulos et al. [28] concerned a community-based sample, compared to the clinical sample of participants in the present study. Moreover, their survey had been conducted in urban and semi-urban areas, where the prevalence of depressive symptoms may be different, as aforementioned. Finally, in that study, a different tool had been used for the ascertainment of depressive symptoms; this was the Geriatric Depression Scale-15. Subsequently, direct comparisons with that study cannot be made. Overall, direct comparisons of the findings of the present study with the limited data from other regions in Greece cannot be made due to differences in methodology, such as measuring instruments and sampling. Age has been correlated with the intensity of depressive symptoms in other studies as well [29]. However, in a study in Brazil, it was found that age was inversely correlated with anxiety; the younger the age, the more the anxiety. Although this finding differs from the results of the present study, it should be noted that in the Brazilian study, the age range of participants was 15–49 years [30].

Another factor that was associated with depressive and anxiety symptoms in the present study was the history of a chronic physical disease. This finding has been consistently reported in the literature, both national [22] and international [30,31,32,33], with very few exceptions [29]. Indeed, it has been argued that a physical illness may act as the life event that triggers a depressive episode in vulnerable individuals. Or, as is the case of stroke and other cardiovascular events, there may exist more specific associations between depression and certain physical disorders [34]. Moreover, there is evidence that depressive symptoms may play a role in the onset, course and outcome of cardiovascular and metabolic disease [35]. More recently, a population-based study in the U.K. found that individuals with self-reported severe or moderately severe depression had a higher risk of several physical conditions, mostly endocrine, musculoskeletal and cardiovascular diseases [36]. Accordingly, the primary healthcare setting, in which all the patients are initially treated, may provide a unique opportunity to inquire for and detect an affective or anxiety syndrome in patients, particularly in elderly women. 

Along with age and the history of a chronic physical disease, other factors were associated with depressive symptoms in the present study as well. Several of those factors have been previously reported in the literature, although not consistently. With regard to the financial status and its correlation with depressive or anxiety symptoms, similar to the present study, a previous study in rural women in Brazil found no significant association [30,37]. Still, other research in rural Afghanistan suggested that participants with depressive symptoms were significantly more likely to have a low-income level [33]. Education has been aversely correlated with symptoms of depression and anxiety in the present study; the higher the educational level, the lower the score of depression symptoms. This finding is in line with previous reports from rural Brazil [31] and rural India [23]. Yet, other research did not identify any association between depressive symptoms and the level of education [29]. With regard to the living status, previous research, similar to the present findings, reported that living alone was a significant predictor for depression in rural women in India [23,38]. Finally, the correlation of not-working status with higher rates of depressive symptoms that was found in the present study is supported by a recent study in rural Afghanistan [33]. It may not be feasible to compare culturally different countries in terms of depression prevalence and the potential sociodemographic correlations; however, it is noteworthy that several factors such as living alone and not working appear to affect women across countries and different settings.

Regarding symptoms of anxiety in rural women, it is noteworthy that in contrast to the present study, the study by Srinivasan et al. [23] in rural India, which used the same assessment instrument (DASS-21), revealed no association between living status and education with such symptoms.

### 4.1. Limitations of the Study

The present study has some limitations that should be considered. The sample size, albeit sufficient for the inquiry of several correlations between symptoms and the examined variables, did not allow for subcategorizing and searching for additional potential correlations. The study sample may not be representative of the rural female population, because it is not population-based, but rather a clinical sample of women that attended the primary mental healthcare setting. It should be noted, however, that most participants visited the study sites for minor, short-term conditions and that women with the most severe chronic diseases had been excluded from the study. Indeed, only 13.2% of participants suffered a chronic physical disease. Additionally, the cross-sectional design of the study does not permit the extraction of any conclusions regarding the medium-term impact of the COVID-19 pandemic on participants’ mental health. The absence of comparable data prior to the pandemic onset also precludes any direct comparisons with the pre-pandemic period. Finally, due to the diversity across studies regarding study design, participants, the instruments used for symptom ascertainment and cultural background, research findings are substantially different across countries. Accordingly, the results of the present study may be more relevant at the national level. Indeed, this study may be relevant for clinical practice in primary healthcare settings in rural Greece, as it is the first relative survey after the pandemic and is focused on women, who are the most vulnerable part of the population in terms of risk for mood and anxiety disorders.

### 4.2. Potential Implications of the Study

It appears that a large number of women attending the primary healthcare setting in rural Greece have clinically relevant symptoms of depression and anxiety. A substantial proportion of those cases may go unnoticed and untreated by primary care professionals if a systematic assessment is not used. Physicians should be aware of the high prevalence of anxiety and depressive syndromes in primary care attendees and the atypical presentation of such syndromes, particularly in the elderly [39]. Conceivably, appropriate training in mental disorders should be assumed for primary care physicians, in order to improve their competancy in diagnosis and the treatment of those disorders. Interestingly, previous research has shown that after intensive education programs, Greek primary care physicians may become efficient in the early diagnosis and effective management of mental disorders [40]. The use of self-rated tools, such as the DASS-21 and other relative instruments, may enable the recognition of potential clinical cases that would require further assessment. Such cases could be referred to locally based, rural community mental health services, such as the mobile mental health units in several regions in Greece [41,42], for further assessment and comprehensive treatment. Referrals of older female patients could be particularly relevant in this regard, given the high rates of depressive and anxiety symptoms in this population and their increased needs in the rural context [43,44]. Self-rated tools may be also used for screening and research in the rural context. 

## 5. Conclusions

The present study highlights the importance of inquiring for symptoms of depression and anxiety in women attending the rural primary care setting. The use of valid and reliable self-reported instruments that are easy to administrate may be helpful in this regard. It appears that such symptoms are highly prevalent, particularly among older women and are related to several previously reported demographic and clinical factors. Whether such symptoms correspond to the medium-term effects of the COVID-19 pandemic on mental health warrants further study. 

## Figures and Tables

**Table 1:** Participants’ demographic and clinical characteristics.

		Ν	N %
Age (years)	18–35	22	17.1%
	36–50	32	24.8%
	51–65	39	30.2%
	>65	36	27.9%
Educational level	Primary education (up to 9 years)	16	12.4%
	Secondary education (10–12 years)	81	62.8%
	Tertiary education (>12 years)	32	24.8%
Living status	Cohabitation	106	82.2%
	Alone	23	17.8%
Employment	Not working	88	68.2%
	Working	41	31.8%
Financial status	Poor	46	35.7%
	Moderate	46	35.7%
	Good	37	28.7%
History of chronic diseases	No	112	86.8%
	Yes	17	13.2%

**Table 2:** Correlations of anxiety and depressive symptoms with demographic and clinical variables.

	Anxiety		Depression	
	Chi-Square	*p*	Chi-Square	*p*
Age	χ^2^(3) = 16.901	0.001	χ^2^(3) = 21.460	<0.001
Education	χ^2^(2) = 9.455	0.009	χ^2^(2) = 9.682	0.008
Living status	χ^2^(1) = 41.448	<0.001	χ^2^(1) = 6.219	0.013
Employment	χ^2^(1) = 16.468	<0.001	χ^2^(1) = 9.076	0.003
Financial status	χ^2^(2) = 0.855	0.652	χ^2^(2) = 0.333	0.847
History of chronic disease	χ^2^(1) = 23.561	<0.001	χ^2^(1) = 48.866	<0.001

## Data Availability

Data are kept in the patients’ charts of the primary healthcare study sites and are confidential.
